# Knock-out of *TERMINAL FLOWER 1* genes altered flowering time and plant architecture in *Brassica napus*

**DOI:** 10.1186/s12863-020-00857-z

**Published:** 2020-05-19

**Authors:** Sukarkarn Sriboon, Haitao Li, Chaocheng Guo, Thaveep Senkhamwong, Cheng Dai, Kede Liu

**Affiliations:** National Key Laboratory of Crop Genetic Improvement, Huazhong Agricultural University, Wuhan, 430070 China

**Keywords:** *TERMINAL FLOWER 1*, PEBP, CRISPR/Cas9, Early flowering, Plant architecture, *Brassica napus*

## Abstract

**Background:**

*TERMINAL FLOWER 1 (TFL1)* is a member of phosphatidylethanolamine-binding protein (PEBP) family, which plays an important role in the determination of floral meristem identity and regulates flowering time in higher plants.

**Results:**

Five *BnaTFL1* gene copies were identified in the genome of *Brassica napus*. The phylogenetic analysis indicated that all five *BnaTFL1* gene copies were clustered with their corresponding homologous copies in the ancestral species, *B. rapa* and *B. oleracea*. The expression of the *BnaTFL1s* were confined to flower buds, flowers, seeds, siliques and stem tissues and displayed distinct expression profiles. Knockout mutants of *BnaC03.TFL1* generated by CRISPR/Cas9 exhibited early flowering phenotype, while the knockout mutants of the other gene copies had similar flowering time as the wild type. Furthermore, knock-out mutants of individual *BnaTFL1* gene copy displayed altered plant architecture. The plant height, branch initiation height, branch number, silique number, number of seeds per silique and number of siliques on the main inflorescence were significantly reduced in the *BnaTFL1* mutants.

**Conclusions:**

Our results indicated that *BnaC03.TFL1* negatively regulates flowering time in *B. napus. BnaC03.TFL1* together with the other *BnaTFL1* paralogues are essential for controlling the plant architecture.

## Background

The transition from vegetative to reproductive stage is strictly controlled by both environmental and developmental signals. Plant needs to achieve a certain stage of developmental competence to respond to environmental factors such as day length (photoperiod), winter temperature (vernalization) and water stress [1]. Endogenous signals such as phytohormones, notably gibberellins, also influence the vegetative to floral transition [2, 3]. Rapeseed (*Brassica napus L*., AACC, 2n = 38) is a major source of edible oil and biofuel*,* which emerged from natural crossing between its progenitors, *B. rapa* (AA, 2n = 20) and *B. oleracea* (CC, 2n = 18) ~ 7500 years ago [4]. Flowering time in rapeseed not only has a crucial impact on yield, but also influences the sowing time of other rotation crops [5]. The timing of flowering and plant requirement for and responsiveness to vernalization are major factors in regional climatic adaptation of elite germplasm. In rapeseed, quantitative trait locus (QTL) analysis has been used to identify candidate flowering time genes. For example, one major QTL related to the *BnaA10.FLC* gene has been demonstrated to be an important regulator that represses winter type rapeseed flowering in winter [6].

In *Arabidopsis*, a model plant for eudicots, flowering time is regulated by the photoperiod, autonomous, vernalization, endogenous gibberellin (GA), age and ambient temperature-dependent pathways [7, 8]. The integrated induction signals from these flowering pathways are transmitted via floral integrator genes such as *FLOWERING LOCUS T* (*FT*), *SUPPRESSOR OF OVEREXPRESSION OF CONSTANS1* (*SOC1*) and *TERMINAL FLOWER1* (*TFL1*) [9–12] to induce the expression of floral meristem identity genes *LEAFY* (*LFY*) and *APETALA1* (*AP1*) at the shoot apical meristem to activate floral transition [13, 14]. The photoperiod pathway regulates flowering time depending on day length [1]. The key regulators in the photoperiod pathway encode proteins with homology to phosphatidylethanolamine binding proteins (PEBPs) [15], which is highly conserved and presented in both animal and plant kingdoms [16]. The PEBP gene family encompasses two particularly important genes, *FT* and *TFL1*, which have been found in *Arabidopsis* and soybean [17–19]. Interestingly, *FT* and *TFL1* share 98% amino acid sequence similarity, but their functions are in antagonistic manners. *FT* is a flowering activator which promotes flowering [19–22], while *TFL1* is a flowering inhibitor and controls the identity of shoot meristem during the plant life span [17, 19, 23, 24].

In *Arabidopsis*, *tfl1* mutant exhibits a shorter vegetative phase, produces fewer leaves, reduces the number of flower buds, branches and flowers and controls the conversion of inflorescence meristem to terminal flower [17, 25, 26]. In contrast, overexpression of the *TFL1* gene promotes secondary inflorescence production and delayed flowering [27, 28]. Similarly, mutation of *CENTRORADIALIS* (*CEN*), an *Antirrhinum TFL1* ortholog, results in the conversion of the normally indeterminate inflorescence to a determinate inflorescence [17, 29]. Both *TFL1* and *CEN* are expressed in the subapical region of the shoot meristem. *TFL1* is expressed in both vegetative and inflorescence shoot meristems, whereas *CEN* is only expressed in the inflorescence meristem [17, 29]. The *Determinate stem* (*Dt1*) mutant exhibited determinate growth and terminal flower formation in soybean [30]. Overexpression of *RCN1* and *RCN2*, the *TFL1* homologs in rice, rendered more branched, denser panicles and delayed transition to reproductive phase [31]. Mutations in *BnaA10.TFL1* have no large effects on flowering time but affect some plant architecture related traits in rapeseed [32, 33] .

Three genome-editing tools have been well developed, including Zinc-Finger Nucleases (ZFNs), Transcription Activator-Like Effector Nucleases (TALENs) and Clustered Regularly Interspaced Palindromic Repeat (CRISPR)-associated protein 9 system (CRISPR/Cas9), which have been widely used in improving traits for productivity and nutrition in crop plants [34]. Among these genome editing tools, CRISPR/Cas9 system is considered the most efficient and simple, which has been rapidly and widely applied for genome editing [35–37]. The CRISPR/Cas9 system has been applied to target mutations in many plant species including *B. napus* [38–47].

In rapeseed, the genetic mechanism underlying the control of flowering time has not been fully understood yet. Thus, one of the objectives of rapeseed breeding is to look for new strategies to alter the flowering behavior. Introducing determinate type of inflorescence in the crop will result in shorter flowering time, earlier and consistent maturation which will greatly facilitate harvest in a short time. There are five paralogs of *TFL1* in the allotetraploid *B. napus* due to the whole genome triplication events occurred in the genomes of *B. rapa* and *B. oleracea*, the two diploid progenitors of *B. napus*. In the present study we aimed to evaluate the role of *TFL1* gene copies in controlling flowering time and plant architecture in *B. napus*. We generated mutants of five paralogues of *TFL1s* in *B. napus* using the CRISPR/Cas9 technology. Knockout mutants of *BnaC03.TFL1* exhibited earlier flowering. In addition, knockout mutant of individual *BnaTFL1* gene copy displayed reduced plant height, branch initiation height, branch number, silique number, seed number per silique and number of siliques on the main inflorescence at different degrees. Our study showed that *BnaC03.TFL1* negatively regulates the conversion of inflorescence meristem to floral meristem in *B. napus*. The *BnaTFL1* gene copies are involved in the determination of plant architecture, and are promising targets for crop improvement in rapeseed.

## Results

### Isolation and identification of *TFL1* genes in *B. napus*

**Fig. 1:**
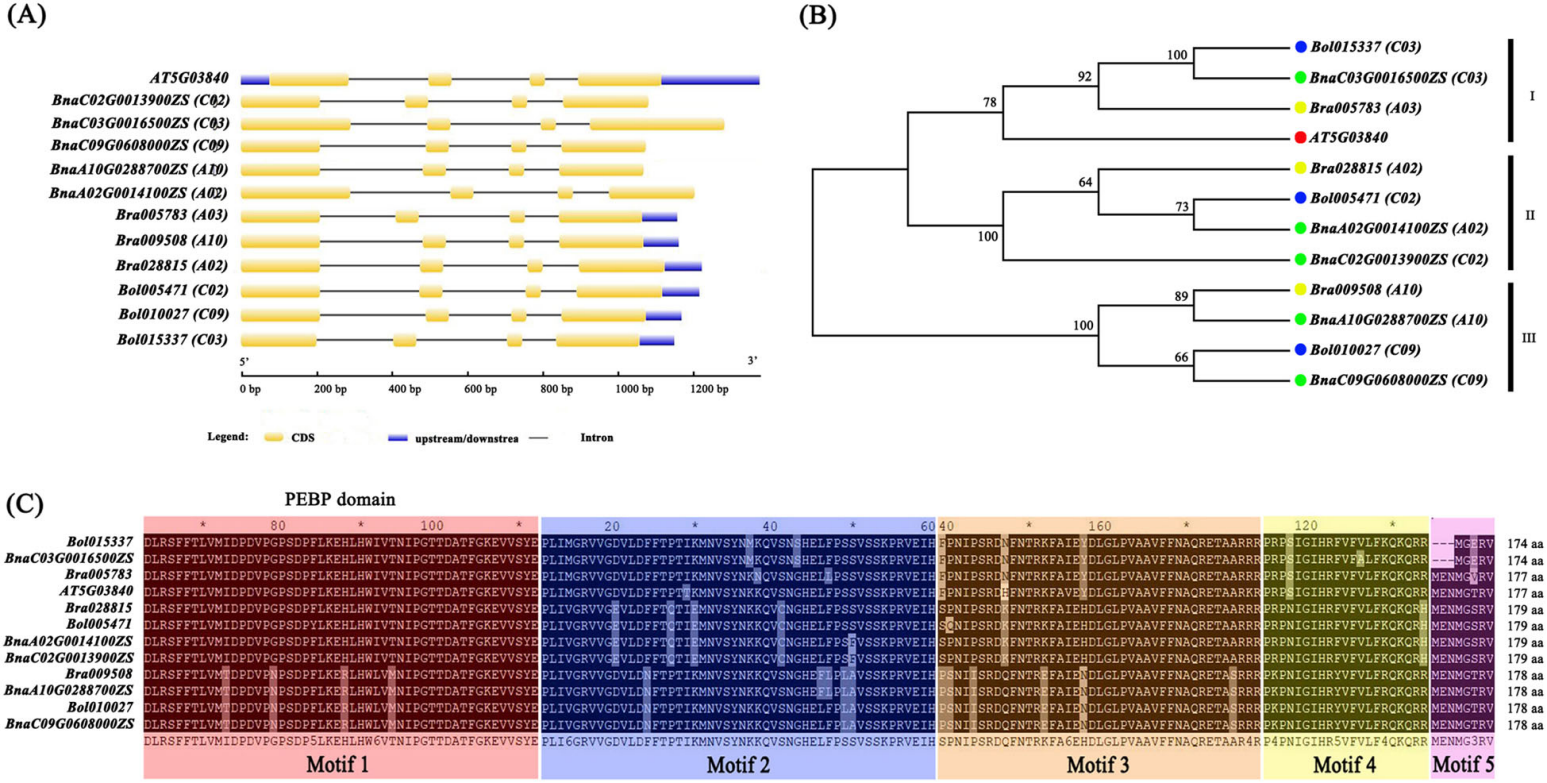
Phylogenetic, gene structure, and domain analyses of *TFL1* genes in *B. napus*. **a** Exon-intron structures of the 12 *TFL1* genes from *B. napus, B. rapa*, *B. oleracea*, and *A. thaliana*. **b** Phylogenetic relationship of the 12 *TFL1* genes. Genes from *B. napus, B. rapa*, *B. oleracea*, and *A. thaliana* were indicated by green, yellow, blue and red circle, respectively. **c** Multiple alignment of amino acid sequences of 12 *TFL1* genes. Motif compositions of PEBP were identified using the MEME tool. Each motif is represented by a colored block. The sizes of amino acid (aa) are shown on the right side of the figure

To retrieve *TFL1* genes from the genomes of *B. napus*, *B. rapa* and *B. oleracea*, the amino acid sequence of *Arabidopsis TFL1* was used as a query to search the *B. napus* (http://rice.hzau.edu.cn/cgi-bin//bnapus/gb2/gbrowse/ZS11v0/) as well as the *B. rapa* and *B. oleracea* databases (http://brassicadb.org/brad/blastPage.php). Five *BnaTFL1* gene copies were obtained, including two (*BnaA02G0014100ZS* and *BnaA10G0288700ZS*) from the A sub-genome, and three (*BnaC02G0013900ZS*, *BnaC03G0016500ZS* and *BnaC09G0608000ZS*) from the C sub-genome. These five gene copies were named as *BnaA02.TFL1*, *BnaA10.TFL1*, *BnaC02.TFL1*, *BnaC03.TFL1* and *BnaC09.TFL1*, respectively. We also obtained three *TFL1* gene copies (*Bra005783*, *Bra028815* and *Bra009508*) from *B. rapa* and three *TFL1* gene copies (*Bol015337*, *Bol005471* and *Bol010027*) from *B. oleracea*. All these gene copies contain four exons, with the lengths of open reading frames (ORFs) ranging from 522 bp to 540 bp (Fig. 1a and Table S2).

The phylogenetic tree was constructed based on their amino acid sequences. The result indicated that *Brassica TFL1* gene copies are divided into three clusters which are consistent with their evolutionary relationship (Fig. 1b). The paralogous gene copies in *B. napus* and their corresponding homologous copies in *B. rapa* and *B. oleracea* are grouped in the same clusters (Fig. 1b). For example, *BnaA02G0014100ZS* and *BnaC02G0013900ZS* are clustered with *Bra028815* from A02 of *B. rapa* and *Bol005471* from C02 of *B. oleracea. BnaC03.TFL1* (*BnaC03G0016500ZS*) and *Arabidopsis TFL1* are cluster I, suggesting that *BnaC03.TFL1* is more closer to *Arabidopsis TFL1* than the other *BnaTFL1s* (Fig. 1b). Multiple alignment of amino acid sequences indicated all the *Brassica TFL1* proteins display very high homology to the *Arabidopsis TFL1*, with identity ranging from 83.4 to 100%. All *TFL1* proteins contain five highly conserved motifs including the PEBP domain, suggesting that these *TFL1* genes may have conserved functions (Fig. 1c).

### Expression pattern of *BnaTFL1*

**Fig. 2:**
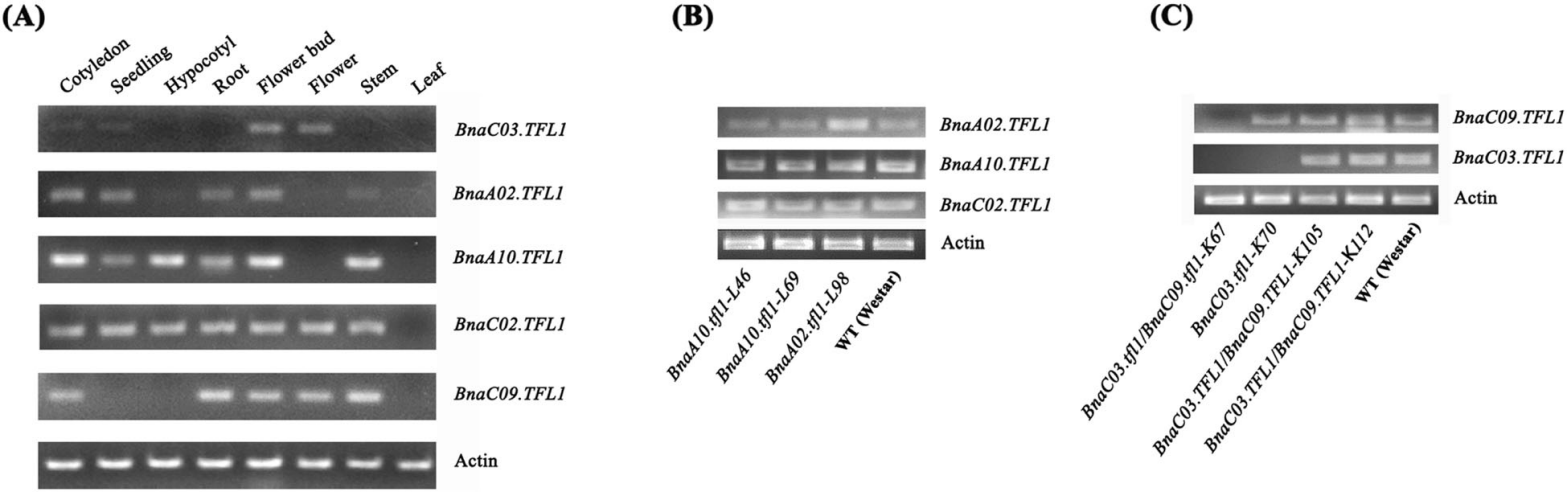
Expression pattern of *BnaTFL1.***a** RT-PCR analyses of *BnaTFL1* genes in wild type Westar. Expression patterns of the five *BnaTFL1s* in cotyledons, seedlings, hypocotyls, roots, flower buds, flowers stem and leaves. **b** The expression of *BnaTFL1s* in flower buds of sgRNA1/2 mutagenized mutants. **c** The expression of *BnaTFL1s* in flower buds of sgRNA3/4 mutagenized mutants. WT stands for wild type Westar

To gain insights into the putative functions of the five *TFL1* gene copies in *B. napus*, we investigated their expression in cotyledons, seedlings, hypocotyls, roots, flower buds, flowers, stems and leaves by RT-PCR. The five *BnaTFL1* gene copies displayed distinct expression patterns (Fig. 2a). All genes are expressed in flower buds but not in leaves. *BnaC02.TFL1* expressed in all tissues except for leaves. *BnaA10.TFL1* and *BnaA02.TFL1* showed similar expression pattern. Both gene copies are expressed in cotyledons, seedlings, hypocotyls, roots, flower buds and stem, but the expression levels of *BnaA02.TFL1* are lower than that of *BnaA10.TFL1* in all these tissues. *BnaC03.TFL1* is preferentially expressed in flower buds and flowers and weakly expressed in cotyledons and seedlings. *BnaC09.TFL1* is expressed in cotyledons, roots, stems, flower buds and flowers but not in seedlings, hypocotyls and leaves (Fig. 2a). Comparison of promoter sequences of these five *BnaTFL1* gene copies indicated that they are divergent, which is consistent with their distinct expression patterns (Fig. S1).

### Targeted mutagenesis of *TFL1* induced by CRISPR/Cas9

**Fig. 3:**
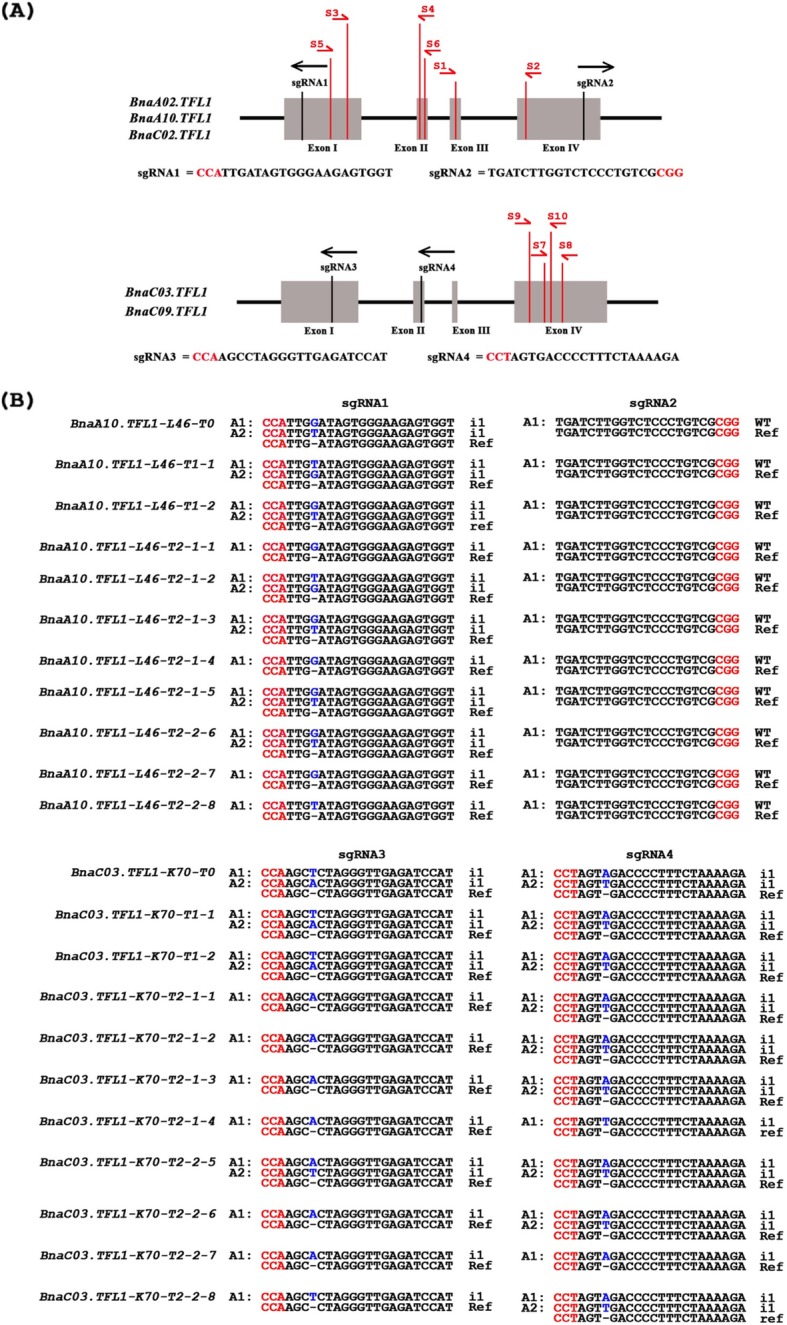
Sequence variations in CRISPR/Cas9-induced *BnaTFL1s* mutants. **a** The structure of *BnaTFL1* genes including four exons (Gray box) separated by three introns (represented by the solid line). The vertical lines in the genes indicate the target sites of sgRNAs, and the arrows indicate directions of the sgRNAs. The red characters indicate the PAM sites in the target sequences. The single-side arrows indicate primers for RT-PCR. The primer pairs of S1/S2, S3/S4, S5/S6, S7/S8 and S9/S10 were used to amplify *BnaC02.TFL1*, *BnaA10.TFL1*, *BnaA02.TFL1*, *BnaC03.TFL1* and *BnaC09.TFL1*, respectively. **b** Sequences variations at the gRNA target sites of *BnaTFL1.A10* (L46) and *BnaTFL1.C03* (K70) in the T0, T1 and T2 generations. Blue characters indicate nucleotide insertions, and i1 means one nucleotide insertion. Ref stands for reference sequence. WT stands for wild-type allele. “A1” and “A2” represent the mutated alleles of the target *Bnatfl1* genes

In order to uncover the functions of these *BnaTFL1* gene copies, the CRISPR/Cas9 gene editing technology was used to create knockout mutants of the five paralogous *BnaTFL1s*. Because we could not identify conserved sgRNAs that can target all five *BnaTFL1* gene copies simultaneously, we designed four sgRNAs to target the five *BnaTFL1s*, with sgRNA1 and sgRNA2 targeting to the conserved regions of *BnaA02.TFL1*, *BnaA10.TFL1* and *BnaC02.TFL1*, and sgRNA3 and sgRNA4 targeting to the conserved regions of *BnaC03.TFL1* and *BnaC09.TFL1* (Fig. 3a). The constructs containing sgRNA1/sgRNA2 and sgRNA3/sgRNA4 were independently transformed into oilseed callus following standard procedures [43]. A total of 101 T0 transgenic plants were obtained for sgRNA1/2 and 130 T0 transgenic plants for sgRNA3/4. Among them, 40 plants were found to be Cas9-positive for sgRNA1/2 transgenic plants, while 60 plants to be Cas9-positive for sgRNA3/4 transgenic plants (Table S3 and S4). Mutations occurred in these five *BnaTFL1* gene copies were screened from these Cas9-positive transgenic plants by ACT-PCR (annealing at critical temperature PCR) [48]. A pair of specific primers were designed for each target sgRNA of the five gene copies and used to screen the Cas9-positive plants. Totally, six T0 plants were identified to have mutations at sgRNA1 and/or sgRNA2 target sites and 12 T0 plants were identified to have mutations at sgRNA3 and/or sgRNA4 target sites by ACT-PCR.

The mutations of individual sgRNA target sites were further confirmed by Sanger sequencing. Among the six T0 mutant plants generated by sgRNA1/2, three plants (L43, L46 and L69) had mutations only in *BnaA10.TFL1*, two plants (L84 and L98) contained mutations only in *BnaA02.TFL1*, and one plant (L93) had mutations in both *BnaA02.TFL1* and *BnaC02.TFL1* (Table S5). No plant had mutations in all three target genes. In these six T0 plants, L46 and L69 were loss-of-function mutants of *BnaA10.TFL1*, and L84 and L98 were loss-of-function mutants of *BnaA02.TFL1.* No loss-of-function mutant was obtained for *BnaC02.TFL1.*

As for the 12 mutant plants generated by sgRNA3/4, five plants (K35, K45, K54, K62 and K85) had mutations in *BnaC09.TFL1*, one plant (K70) had mutation in both *BnaC03.TFL1*, and the others (K67, K74, K87, K105, K112 and K130) had mutations in *BnaC03.TFL1* and *BnaC09.TFL1* (Table S6). In these mutant plants, K70 was a loss-of-function mutant of *BnaC03.TFL1*, and K67 and K87 were loss-of-function mutants of both *BnaC03.TFL1* and *BnaC09.TFL1*. K85 was a loss-of-function mutant of *BnaC09.TFL1*. K105 and K112 were chimeric containing wild type alleles.

### Detection of CRISPR/Cas9 mediated mutations of *BnaTFL1* in the T1 and T2 generations

To assess the inheritance of the CRISPR/Cas9-induce mutations in next generations, we self-pollinated two *BnaA10.TFL1* mutants (L46 and L69), one *BnaA02.TFL1* mutant (L98), one *BnaC03.TFL1* mutant (K70) and one *BnaC09.TFL1* mutant (K85), three *BnaC03.TFL1/BnaC09.TFL1* double mutants (K67, K105 and K112) to obtain their T1 generation. Two T1 plants were randomly selected from each line to check their genotypes by TA cloning followed by Sanger sequencing. The two T1 plants derived from these eight T0 mutants had the same mutations as their parents (Table S7), except for K105 and K112 which generated new mutations in T1 due to the existence of wild-type allele in the T0 plants.

The transmission of mutations from T1 to T2 generation was further investigated by next-generation sequencing of target amplicons. Eight T2 plants were randomly selected from one of the T1 plants. Each target site was amplified using target-specific primers and amplicons were sequenced using Illumina HiSeq 4000 sequencing platform. The T2 plants of L46, L69, L98, K67 and K70 had the same mutations as their parents, and all were loss-of-function mutants with homozygous or heterozygous genotypes of mutant alleles (Table S8 and S9). For examples, four T2 plants of L46 were heterozygous of two mutant alleles (bi-allelic, i1–1/i1–2) and four T2 plants were homozygous (i1–1/i1–1). Of the eight T2 plants of K70, seven were homozygous (i1–1/i1–1) and one was heterozygous (bi-allelic, i1–1/i1–2). Same results were found in other mutant lines (Fig. 3b). All the T2 plants of K85 were chimeric of multiple mutant alleles, while all the T2 plants of K105 and K112 were chimeric of multiple mutant alleles and wild-type allele. These results indicated that most mutations were inheritable from T1 to T2 generations (Tables 1 and 2).

**Table 1:** Genotypic analysis of *BnaTFL1s* mutants (*BnaA02.TFL1*, *BnaA10.TFL1*, and *BnaC02.TFL1.*) mutagenized by sgRNA1/2 in the T1 and T2 generations

Lines	Generations	***BnaA02.TFL1***		***BnaA10.TFL1***		***BnaC02.TFL1***		
		sgRNA1	sgRNA2	sgRNA1	sgRNA2	sgRNA1	sgRNA2	Early flowering
L46	T0	WT	WT	Bi-allelic (i1–1/i1–2)	WT	WT	WT	No
L46–1	T1	WT	WT	Bi-allelic (i1–1/i1–2)	WT	WT	WT	No
L46–1-1	T2	WT	WT	Homozygous (i1–1/i1–1)	WT	WT	WT	No
L46–1-2	T2	WT	WT	Bi-allelic (i1–1/i1–2)	WT	WT	WT	No
L46–1-3	T2	WT	WT	Bi-allelic (i1–1/i1–2)	WT	WT	WT	No
L46–1-4	T2	WT	WT	Homozygous (i1–1/i1–1)	WT	WT	WT	No
L69	T0	WT	WT	Bi-allelic (i1–1/i1–2)	WT	WT	WT	No
L69–1	T1	WT	WT	Bi-allelic (i1–1/i1–2)	WT	WT	WT	No
L69–1-1	T2	WT	WT	Homozygous (i1–1/i1–1)	WT	WT	WT	No
L69–1-2	T2	WT	WT	Bi-allelic (i1–1/i1–2)	WT	WT	WT	No
L69–1-3	T2	WT	WT	Bi-allelic (i1–1/i1–2)	WT	WT	WT	No
L69–1-4	T2	WT	WT	Homozygous (i1–1/i1–1)	WT	WT	WT	No
L98	T0	WT	Homozygous (d1–1/d1–1)	WT	WT	WT	WT	No
L98–1	T1	WT	Homozygous (d1–1/d1–1)	WT	WT	WT	WT	No
L98–1-1	T2	WT	Homozygous (d1–1/d1–1)	WT	WT	WT	WT	No
L98–1-2	T2	WT	Homozygous (d1–1/d1–1)	WT	WT	WT	WT	No
L98–1-3	T2	WT	Homozygous (d1–1/d1–1)	WT	WT	WT	WT	No
L98–1-4	T2	WT	Homozygous (d1–1/d1–1)	WT	WT	WT	WT	No

*d1* one nucleotide deletion; *i1* one nucleotide insertion; *WT* wild type; i1–1 and i1–2 stand for two different alleles

**Table 2:** Genotypic analysis of *BnaTFL1s* mutants (*BnaC03.TFL1* and *BnaC09.TFL1*) mutagenized by sgRNA3/4 in the T1 and T2 generations

Lines	Generations	***BnaC03.TFL1***		***BnaC09.TFL1***		
		sgRNA3	sgRNA4	sgRNA3	sgRNA4	Early flowering
K67	T0	Bi-allelic (d1/d5)	Chimeric (i1–1/i1–2/WT)	Bi-allelic (i1–1/i1–2)	WT	Yes
K67–1	T1	Bi-allelic (d1/d5)	Chimeric (i1–1/i1–2/WT)	Bi-allelic (i1–1/i1–2)	WT	Yes
K67–1-1	T2	Bi-allelic (d1/d5)	Chimeric (i1–1/i1–2/WT)	Homozygous (i1–1/i1–1)	WT	Yes
K67–1-2	T2	Bi-allelic (d1/d5)	Chimeric (i1–1/i1–2/WT)	Homozygous (i1–1/i1–1)	WT	Yes
K67–1-3	T2	Bi-allelic (d1/d5)	Chimeric (i1–1/i1–2/WT)	Bi-allelic (i1–1/i1–2)	WT	Yes
K67–1-4	T2	Bi-allelic (d1/d5)	Chimeric (i1–1/i1–2/WT)	Homozygous (i1–1/i1–1)	WT	Yes
K70	T0	Bi-allelic (i1–1/i1–2)	Bi-allelic (i1–1/i1–2)	WT	WT	Yes
K70–1	T1	Bi-allelic (i1–1/i1–2)	Bi-allelic (i1–1/i1–2)	WT	WT	Yes
K70–1-1	T2	Homozygous (i1–1/i1–1)	Bi-allelic (i1–1/i1–2)	WT	WT	Yes
K70–1-2	T2	Homozygous (i1–1/i1–1)	Bi-allelic (i1–1/i1–2)	WT	WT	Yes
K70–1-3	T2	Homozygous (i1–1/i1–1)	Bi-allelic (i1–1/i1–2)	WT	WT	Yes
K70–1-4	T2	Homozygous (i1–1/i1–1)	Homozygous (i1–1/i1–1)	WT	WT	Yes
K85	T0	WT	WT	Chimeric (i1–1/i1–2/d1/d5)	WT	No
K85–1	T1	WT	WT	Chimeric (i1–1/i1–2/i1–3)	WT	No
K85–1-1	T2	WT	WT	Chimeric (i1–1/i1–2/i1–3/d1)	WT	No
K85–1-2	T2	WT	WT	Chimeric (i1–1/i1–2/i1–3/d1)	WT	No
K85–1-3	T2	WT	WT	Chimeric (i1–1/i1–2/i1–3/d1)	WT	No
K85–1-4	T2	WT	WT	Chimeric (i1–1/i1–2/d1)	WT	No
K105	T0	Chimeric (i1–1/i1–2/i1–3/WT)	WT	Chimeric (i1–1/i1–2/i1–3/i1–4/WT)	WT	No
K105–1	T1	Chimeric (i1/4d/WT)	WT	Chimeric (i1–1/i1–2/i1–3/WT)	WT	No
K105–1-1	T2	Chimeric (i1–1/i1–2/i1–3/WT)	WT	Chimeric (i1–1/i1–2/i1–3/WT)	WT	No
K105–1-2	T2	Chimeric (i1–1/i1–2/i1–3/WT)	WT	Chimeric (i1–1/i1–2/i1–3/WT)	WT	No
K105–1-3	T2	Chimeric (i1–1/i1–2/i1–3/WT)	WT	Chimeric (i1–1/i1–2/i1–3/WT)	WT	No
K105–1-4	T2	Chimeric (i1–1/i1–2/i1–3/WT)	WT	Chimeric (i1–1/i1–2/WT)	WT	No
K112	T0	Chimeric (i1–1/i1–2/5d)	WT	Chimeric (i1–1/i1–2/i1–3)	WT	No
K112–1	T1	Chimeric (i1/d5/WT)	WT	Chimeric (i1–1/i1–2/i1–3/WT)	WT	No
K112–1-1	T2	Chimeric (i1–1/i1–2/i1–3/WT)	WT	Chimeric (i1–1/i1–2/i1–3/WT)	WT	No
K112–1-2	T2	Chimeric (i1–1/i1–2/WT)	WT	Chimeric (i1–1/i1–2/i1–3/WT)	WT	No
K112–1-3	T2	Chimeric (i1–1/i1–2/WT)	WT	Chimeric (i1–1/i1–2/i1–3/WT)	WT	No
K112–1-4	T2	Chimeric (i1–1/i1–2/WT)	WT	Chimeric (i1–1/i1–2/i1–3/WT)	WT	No

d1, d4 and d5 means one, four and five nucleotides deletion, respectively; *i1* one nucleotide insertion; *WT* wild type; i1–1, i1–2, etc. stands for different alleles

To check the expression of the target genes in their corresponding mutants, we performed RT-PCR to detect their mRNA transcripts in flower buds where all the five *BnaTFL1* gene copies are expressed. The expression levels of *BnaA02.TFL1*, *BnaA10.TFL1* and *BnaC02.TFL1* did not show significant changes in the knockout mutant lines L98 (*BnaA02.tfl1*), and L46 and L69 (*BnaA10.tfl1*) created by sgRNA1/2, suggesting that the insertions or deletions did not affect the transcription of these genes (Fig. 2b). We also checked the expression of *BnaC03.TFL1* and *BnaC09.TFL1* in flower buds of different mutants induced by sgRNA3/4 and found *BnaC03.TFL1* was not detectable in single gene knockout mutant *BnaC03.tfl1* (K70) and in double mutant *BnaC03.tfl1/BnaC09.tfl1* (K67). *BnaC09.TFL1* was not detectable in the double mutant *BnaC03.tfl1/BnaC09.tfl1* (K67) (Fig. 2c). These results suggested that insertions or deletions abolish the expression of these two genes. The expression levels of *BnaC03.TFL1* and *BnaC09.TFL1* were not affected in the chimeric mutant lines K105 and K112 having wild-type alleles (Fig. 2c).

#### Knockout of *BnaC03.TFL1* promotes flowering in *B. napus*

**Fig. 4:**
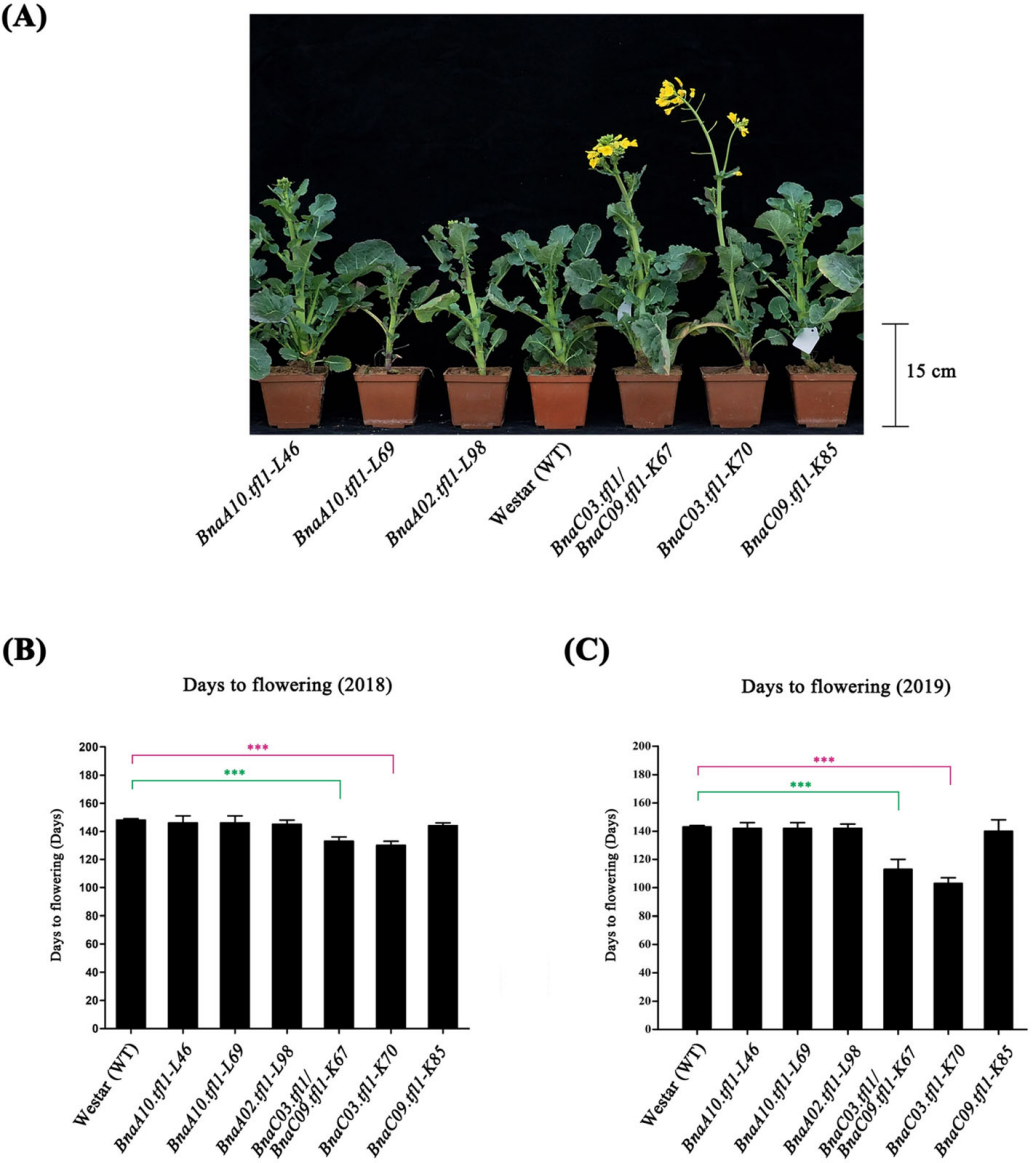
Flowering time of CRISRP/Cas9-induced *BnaTFL1s* mutants. **a** Morphology and flowering time of Westar and CRISRP/Cas9-induced *BnaTFL1s* mutants. **b** Comparison of flowering time between the WT and *BnaTFL1s* mutants in the T1 generation (2018). **c** Comparison of flowering time between the WT and *BnaTFL1s* mutants in the T2 generation (2019). DTF stands for days from sowing to flowering. Statistically significant differences were revealed using Student’s *t*-test: *, *P* < 0.05; **, *P* < 0.01, and ***, *P* < 0.001. Values are means ± SD (*n* = 20)

It is reported that *Arabidopsis TFL1* negatively regulates flowering time [17, 23]. To investigate if the knockout of *BnaTFL1* gene copies affect the flowering time, we grew the *Bnatfl1* mutants in the field under nature growth conditions in two growth seasons (2018 for T1, and 2019 for T2) to observe the agronomic traits. In the T1 generation, single mutants including *BnaA10.tfl1* (L46 and L69), *BnaC09.tfl1* (K85) and *BnaA02.tfl1* (L98) had the same days from sowing to flowering (DTF) as WT (Westar) (Fig. 4a, b and Table S10). Among the four T1 mutant lines having mutations in *BnaC03.TFL1*, K70 (130 ± 3d) for *BnaC03.tfl1* and K67 (133 ± 3d) for *BnaC03.tfl1/BnaC09.tfl1* flowered much earlier than Westar (Fig. 4a and b). K85 was a loss-of-function mutant at *BnaC09.TFL1* and displayed normal flowering time as wild type Westar (Fig. 4a, b and Table S10). In the T2 generation, the flowering time of these mutants was similar to that in the T1 generation (Fig. 4c and Table S11). The results indicated that the loss-of-function mutants of *BnaC03.TFL1* exhibited early flowering and the loss-of-function mutants of the other gene copies did not affect the flowering time, suggesting that *BnaC03.TFL1* play an important role in determining the floral transition, while the other paralogues may have obtained new functions or function redundantly in the determination of floral transition.

### *BnaTFL1* genes regulate plant architecture in *B. napus*

**Fig. 5:**
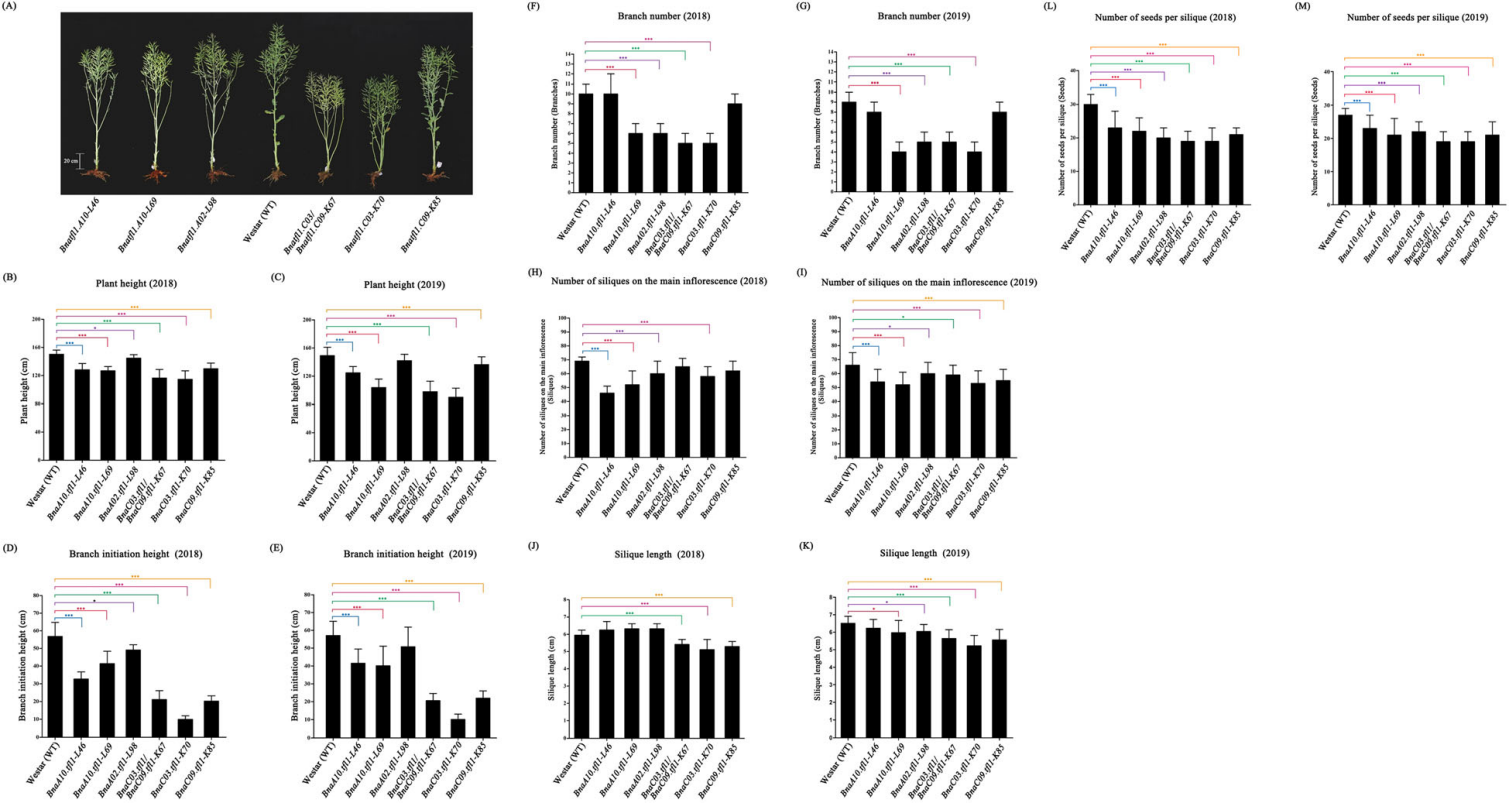
Differences of plant architecture related traits in CRISRP/Cas9-induced *BnaTFL1s* mutants. **a** Whole plant architecture of T2 plants of CRISRP/Cas9-induced *BnaTFL1s* mutants. Differences of plant height (**b** and **c**), Branch initiation height (**d** and **e**), Branches number (**f** and **g**), Number of siliques on the main inflorescence (**h** and **i**), Silique length (**j** and **k**), and Number of seeds number per siliques (**l** and **m**) in 2018 (left panel) and 2019 (right panel). Statistically significant differences were revealed using Student’s *t*-test: *, *P* < 0.05; **, *P* < 0.01, and ***, *P* < 0.001. Values are means ± SD (*n* = 20)

*Arabidopsis TFL1* also determines the formation of shoot apical meristem and thus the plant architecture. *tfl1* mutants displayed altered plant architecture such as less leaves, branches and flowers than the wild type. To see if the *BnaTFL1* gene copies also regulate plant architecture in rapeseed, we investigated plant height (PH) and branch initiation height (BIN) of these single mutants and double mutants in the T1 and T2 generations (2018 and 2019, respectively). The knockout mutants of *BnaA10.TFL1*, *BnaC03.TFL1* and *BnaC09.TFL1* were significantly shorter than WT (150.2 ± 5 cm in 2018 and 149.1 ± 12 cm in 2019) (Fig. 5b and c). Consequently, the branch initiation height of these knockout mutants were also significantly lower than that of the wild type (Fig. 5d and e). Of these knockout mutants, *BnaC03.tfl1* (K70) displayed the largest reduction in both plant height and branch initiation height. These results suggested that these four *BnaTFL1s* are involved in the determination of plant height, with *BnaC03.TFL1* having the strongest and *BnaA02.TFL1* having the weakest effects on determining the plant height and the position of the first branch.

We also investigated four yield-related traits including branch number (BN), number of siliques on the main inflorescence (NSMI), siliques length (SL), and number of seeds per silique (NSS) of these mutants in the T1 and T2 generations (2018 and 2019, respectively). We also noticed that *BnaA10.tfl1* (L69), *BnaA02.tfl1* (L98), *BnaC03.tfl1* (K70) and *BnaC03.tfl1/BnaC09.tfl1* (K67) had much less branches than the wild type, while *BnaC09.tfl1* had similar number of branches to the wild type (Fig. 5f and g). In addition, all the knockout mutants had less siliques on the main inflorescence (NSMI), shorter siliques and less seeds per silique than the wild type (Fig. 5h - m). These results indicated that the yield-related traits in all the *Bnatfl1s* mutants were affected at different degrees, suggesting that these *BnaTFL1* gene copies play roles in the determination of plant architecture.

## Discussions

*Arabidopsis* has only one *TFL1* gene which functions in the control of flowering time and floral architecture. While polyploids or paleopolyploid species usually have multiple *TFL1* gene copies with distinct expression patterns and divergent functions in the control of flowering time and floral architecture [49]. Of three *TFL1* homologs isolated in pea, *PsTFL1a* corresponds to *DETERMINATE* (*DET*) gene and *PsTFL1c* corresponds to the *LATE FLOWERING* (*LF*) gene. *DET* specifically expresses in the shoot apex after floral initiation and acts to maintain the indeterminacy of the apical meristem during flowering, while *LF* controls the length of the vegetative phase by delaying floral initiation [50]. In this study, we identified five *BnaTFL1* gene copies in the genome of allotetraploid *B. napus*. These *BnaTFL1* gene copies together with *TFL1* gene copies from the ancestor species *B. rapa* and *B. oleracea* were classified into three clusters (Fig. 1b). The five *BnaTFL1s* originated from their corresponding ancestral gene copies in *B. rapa* and *B. oleracea* and had distinct expression patterns, suggesting that they may have different roles in regulating the flowering time and/or maintaining the inflorescence meristem in *B. napus*. In the knockout mutants, only *BnaC03.tfl1* single mutant and *BnaC03.tfl1*/*BnaC09.tfl1* double mutant exhibited earlier flowering, indicating that *BnaC03.TFL1* is involved in regulating flowering time (Fig. 4) [17, 23]. And consistent with this role, *BnaC03.TFL1* is preferentially expressed in flower bud and flower tissues (Fig. 2a). In the double mutant *BnaC03.tfl1/BnaC09.tfl1*, *BnaC09.tfl1* did not enhance the early flowering phenotype of *BnaC03.tfl1* (Fig. 4a, b and c), suggesting that *BnaC09.TFL1* is not redundant to *BnaC03.TFL1* because double mutants usually have stronger phenotypes than single mutants. Except for *BnaC03.tfl1* and *BnaC03.tfl1*/*BnaC09.tfl1* mutants, the knockout mutants including *BnaA02.tfl1*, *BnaA10.tfl1* and *BnaC09.tfl1* did not exhibit early flowering, suggesting that these *BnaTFL1* gene copies may be not involved in the control of flowering time. However, we could not exclude the possibility that *BnaA02.TFL1*, *BnaA10.TFL1* and *BnaC09.TFL1* redundantly regulate flowering time. Unfortunately, we failed to obtain single mutant of *BnaC02.TFL1*, and the double and triple mutants of *BnaA10.TFL1*, *BnaA02.TFL1*, and *BnaC02.TFL1*, and their redundancy in controlling flowering time are needed to further investigate.

In addition to early flowering, the knockout mutants of *BnaC03.TFL1* (K67 and K70) also displayed altered plant architecture. The plant height, branch initiation height and yield related traits including branch number, silique length, number of seeds per silique and number of siliques on the main inflorescence in the *BnaC03.TFL1* mutants were significantly reduced when compared to the wild type. These results demonstrated that, similar to *Arabidopsis TFL1*, *BnaC03.TFL1* plays important roles in determining flower time and plant architecture, which is consistent with the phylogenetic tree in which *BnaC03.TFL1* is more closely related to *Arabidopsis TFL1* (Fig. 1b). However, we could not exclude that the alteration of plant architecture in *BnaC03.tfl1* and *BnaC03.tfl1/BnaC09.tfl1* is caused by altered flowering time. Besides *BnaC03.TFL1*, the mutation of *BnaA02.TFL1*, *BnaA10.TFL1* and *BnaC09.TFL1* also altered plant architecture. The plant height, branch initiation height and yield related traits including number of branches, number of siliques on the main inflorescence, silique length and number of seeds per silique in these *BnaTFL1s* mutants were decreased at different degrees when compared to the wild type. In all of the *BnaTFL1s* mutants, *BnaC03.tfl1* exhibited the strongest alteration of plant architecture. The altered plant architecture was also observed in *BnaA10.tfl1* mutants in previous studies [32, 33]. An EMS mutant of *BnaA10.TFL1* has altered yield component traits [32]. *Bnsdt1*, a natural mutant of *BnaA10.TFL1*, has reduced plant height, but displayed normal flowering time, yield and yield-related traits including silique number, silique density and seeds per silique as the wild type [33, 51]. It is speculated that the lowered expression of *BnaA10.TFL1* in *Bnsdt1* results in the determinate inflorescence but does not affect yield-related traits and seed yield [33, 51]. These results indicated that *BnaA02.TFL1*, *BnaA10.TFL1* and *BnaC09.TFL1*, together with *BnaC03.TFL1*, are important key regulatory genes involved in the control of floral architecture. Although the knockout mutants of *BnaTFL1s* in this study had deteriorated yield-related traits, it provides us promising targets for plant architecture improvement in rapeseed. We could manipulate the expression level of these *BnaTFL1s* to obtain mutant lines with determinate inflorescence and similar or better yield and yield-related trait performance as the natural variation *Bnsdt1* [51].

## Conclusions

In this study, we identified five *TFL1* gene copies in the *B. napus* genome. These gene copies display different expression patterns, suggesting that the functions of the *BnaTFL1* genes have sub-functionalized. Knock-out mutants generated by CRISPR/Cas9 technology indicated that *BnaC03.TFL1* negatively regulates flowering time in *B. napus.* In addition, all the *BnaTFL1* gene copies are involved in the control of plant architecture related traits including plant height, branch initiation height and branch number. Our findings provide a base for future understanding the functions of *BnaTFL1* genes and for modification of plant architecture of rapeseed.

## Methods

### Identification of *TFL1* genes in *Brassica* species

The sequences of *Arabidopsis TFL1* gene was obtained from the TAIR database (http://www.arabidopsis.org) and used as queries for a BLASTP algorithm-based against the *B. napus* database (http://rice.hzau.edu.cn/cgi-bin//bnapus/gb2/gbrowse/ZS11v0/), and *Brassica* database (http://brassicadb.org/brad/blastPage.php) with a *p*-value cutoff of 0.001 to retrieve homologous genes from the *B. napus*, *B. rapa*, and *B. oleracea*, respectively. For all candidate genes, we also examined whether they contain the PEBP domain in the SMART (http://smart.embl-heidelberg.de) and Pfam (http://pfam.sanger.ac.uk) databases. Sequences without a PEBP domain were deleted. Protein information including the length of amino acids (a.a.) was examined using the online web tool Expasy (ProtParam) (https://web.expasy.org/protparam/).

### Conserved motifs and gene structure analysis of *TFL1* proteins in *Brassica* species

Conserved motifs in *TFL1* proteins of *Brassica* species were identified using the program SMART (Pfam) (http://smart.embl-heidelberg.de/), HMMAR and MEME (http://meme-suite.org/) by using default parameters [52]. The *TFL1* gene structure was predicted using the program of GSDS2.0 (Gene Structure Display Server, http://gsds.cbi.pku.edu.cn/) for both genome and coding domain sequences.

### Phylogenetic tree construction and protein conserved domain sequence alignment

The *TFL1* amino acid sequences of *B. napus* together with *A. thaliana*, *B. rapa* and *B. oleracea* were aligned using Clustalx 2.0 with default settings. The phylogenetic tree was constructed with MEGA7.0 software using the neighbor-joining method [53]. The bootstrap test was executed by 1000 replications. The resulting phylogenetic tree was prepared in MEGA7.0 software.

### Vector construction and plant transformation

The genomic sequence of *BnaTFL1* genes were subjected to the online CRISPR-P software (http://crispr.hzau.edu.cn/CRISPR2/) to search for guide RNA (gRNA) targets. Two gRNAs were designed in the conserved phosphatidylethanolamine-binding protein (PEBPs) domain for each *TFL1* gene. The gRNA sequences (20 bp) were followed by NGG (PAM, protospacer adjacent motif) at the 3′ end of the forward or reverse strands. Finally, the two AtU6 promoter-sgRNA-AtU6 terminator cassettes in template plasmid pCBC-DT1T2 were amplified using the primers shown in Table S1. The PCR fragments were inserted into the pKSE401 vector by Golden Gate Assembly [54].

For plant transformation, a commonly used *B. napus* cultivar, Westar (An original Canada rapeseed cultivar introduced by our lab) was used as the transformation host in this study [55]. The agrobacterium strain, GV3101 harboring pKSE401-sgRNA vector containing sgRNAs was used to infect the hypocotyls of *B. napus* as previously described [43]. The transgenic calli resistant to kanamycin (50 mg/ml) were allowed to grow until the development of roots and shoots under controlled temperature of 23 °C and a photoperiod of 16 h/8 h (day/night). The kanamycin resistant plants (Kan+) were then transferred to soil in the greenhouse for seed harvesting.

### ACT-PCR assay

The ACT-PCR assay was performed as previous described [48]. One primer target on the sgRNA sites, and the other primer targets on the gene specific region. Genomic DNA was extracted from young leaves of transgenic T0 plants using the cetyltrimethyl ammonium bromide (CTAB) method. The positive transgenic lines were first screened by Cas9 specific primers. Then, the ACT-PCR program was followed as 94 °C for 5 min, 35 cycles at 94 °C for 30 s, gradient 60 °C to 66 °C for 30 s, and 72 °C for 40 s, and 10 min at 72 °C for final extension. All primers were listed in Table S1.

### The genotyping of transgenic lines

To analyze the mutations caused by CRISPR/Cas9, genomic DNA was extracted from each transgenic plant using the CTAB method. The flanking sequence around the CRISPR target sites was amplified by PCR using gene-specific primers. In the T0 and T1 generations, most of the amplicons were directly sequenced to analyze the mutations by DSDECODE (http://skl.scau.edu.cn/dsdecode). For the complex mutations, the amplicons were first sub-cloned into the pGEM-T easy vector, and about 10 clones of each amplicon were individually sequenced by Sanger sequencing. All primers were listed in Table S1.

In the T2 generation, the mutation sites were genotyped by next-generation sequencing of target amplicons [56], which includes two rounds of PCR amplification. The first round PCR profiles were 94 °C for 5 min, 30 cycles at 94 °C for 30 s, gradient 57 °C to 59 °C for 30 s, and 72 °C for 40 s, and 10 min at 72 °C for final extension. The second round profiles were 95 °C for 3 min, 20 cycles at 95 °C for 15 s, 65 °C for 30 s, and 72 °C for 15 s, and 5 min at 72 °C for final extension. All primers were listed in Table S1. PCR products were sequenced by the GenoSeq Company, Wuhan, China.

### Survey of flowering time and architecture traits

The flowering time of the T1 and T2 mutant plants were recorded as the days from the sowing to the appearance of first flower on the main inflorescence. Flowering time and architecture traits including plant height (PH), branches number (BN), branch initiation height (BIN), number of siliques on the main inflorescence (NSMI), siliques length (SL), and number of seeds per siliques (NSS) were measured with at least 20 plants per mutant line. Plant height (PH) was measured as the length of the plant from the base of the stem to the tip of the main inflorescence. Branches number (BN) was measured as all the number of branches arising from the main inflorescence. Branch initiation height (BIN) was measured as the length from the base of the stem to the first primary branch base. Numbers of siliques on the main inflorescence (NSMI) was measured all siliques on the main inflorescence. Siliques length (SL) and number of seeds per siliques (NSS) were measured based on twenty well-developed siliques from of the main inflorescence. Statistical analyses were performed using the student’s *t*-test with R-software to compare the differences of phenotypes between the mutant and wild-type plants at the *P* ≤ 0.05, *P* ≤ 0.01 and *P* ≤ 0.001probability levels.

### RNA extraction and RT-PCR

Total RNA from different plant tissues were extracted using TRIzol reagent (Aidlab, Wuhan, China) following the manufacturer’s instructions. Approximately 2 μg of total RNA was used for cDNA synthesis using a PrimeScript RT reagent kit (ThermoFisher Scientific). Reverse transcription PCR (RT-PCR) programme was 94 °C for 3 min, 35 cycles at 94 °C for 30 s, 60 °C for 30 s, and 72 °C for 30 s, and 5 min at 72 °C for final extension. The *BnActin* gene (GenBank: AF111812.1) served as the internal control. All primers were listed in Table S1.

## Supplementary information

**Additional file 1 Table S1** List of PCR primers and their applications.

**Additional file 2 Table S2** list of 12 *TFL1* genes identified in *B. napus*, *B. oleracea*, *B. rapa*, and *A. thaliana*.

**Additional file 3 Table S3** Percentage of mutated plants in the T0 generation in *Brassica napus*.

**Additional file 4 Table S4** Percentage of mutated plants in the T0 generation in *Brassica napus*.

**Additional file 5 Table S5** The mutation of sgRNAs (sgRNA1 and sgRNA2) of T0 plants found with mutations in the target sequence.

**Additional file 6 Table S6** The mutation of sgRNAs (sgRNA3 and sgRNA4) of T0 plants found with mutations in the target sequence.

**Additional file 7 Table S7** The detection of CRISPR/Cas9 mediated mutations in the T1 generation.

**Additional file 8 Table S8** The detection of CRISPR/Cas9 mediated mutations in the T2 generation.

**Additional file 9 Table S9**. Genotypic analysis of *BnaTFL1* mutants and their transmission to T1 and T2 generations.

**Additional file 10 Table S10** The survey and student t-test resulted for days to flowering and architecture of *bnatfl1* in T1 (2018) generation.

**Additional file 11 Table S11** The survey and student t-test resulted for days to flowering and architecture of *bnatfl1* in T2 (2019) generation.

**Additional file 12 Fig. S1** The promoter sequences alignment between five *BnaTFL1s*; *BnaTFL1.A02*, *BnaTFL1.A10*, *BnaTFL1.C02*, *BnaTFL1.C03* and *BnaTFL1.C09*.

## Data Availability

The data sets supporting the results of this article are included in this manuscript and its additional information files.

## Abbreviations

TFL1: Terminal Flower 1
FT: Flowering Locus T
SOC1: Suppressor of Overexpression of Constans1
FLC: Flowering Locus C
LEF: Leafy
AP1: Apetala1
Cen: Centroradialis
RCN1: Rice Centroradialis 1
RCN2: Rice Centroradialis 2
Dt1: Determinate stem
DET: Determinate
LF: Late flowering
*Bnsdt1*: *Brassica napus* Determinate
*Bna*: *Brassica napus*
*Bra*: *Brassica rapa*
*Bol*: *Brassica oleracea*
GA: Gibberellin acid
QTL: Quantitative trait locus
PEBPs: Phosphatidylethanolamine binding proteins
MEGA: Molecular Evolutionary Genetics Analysis
GSDS: Gene Structure Display Server
Pfam: Protein families database
CRISPR/Cas9: Clustered Regulatory Interspersed Short Palindromic Repeat associated protein
SMART: Simple Modular Architecture Research Tool
TAIR: The Arabidopsis Information Resource
aa: Amino acid
MEME: Multiple Expectation Maximization for Motif Elicitations
ACT-PCR: Annealing at critical temperature Polymerase Chain Reaction
sgRNA: Single-guide RNA
CTAB: Cetyl trimethylammonium bromide
DNA: Deoxyribonucleic acid
mRNA: Messenger RNA
DTF: Days to flowering
PH: Plant height
BN: Branches number
BIN: Branch initiation height
NSMI: Number of siliques on the main inflorescence
SL: Silique length
NSS: Number of seed per siliques
WT: Wild type
DH: Double haploid
RT-PCR: Reverse transcription polymerase chain reaction
ORFs: Open reading frames
EMS: Ethyl methanesulfonate
