# Supplementary material for: Knock-out of TERMINAL FLOWER 1 genes altered flowering time and plant architecture in Brassica napus
Source: BMC Genet. 2020 May 19;21:52. doi: 10.1186/s12863-020-00857-z (PMC7236879; doi:10.1186/s12863-020-00857-z)
Supplement: Supplementary file 12 — Additional file 12 Fig. S1 The promoter sequences alignment between five BnaTFL1s; BnaTFL1.A02, BnaTFL1.A10, BnaTFL1.C02, BnaTFL1.C03 and BnaTFL1.C09. [file 12863_2020_857_MOESM12_ESM.pptx]

## Slide 1
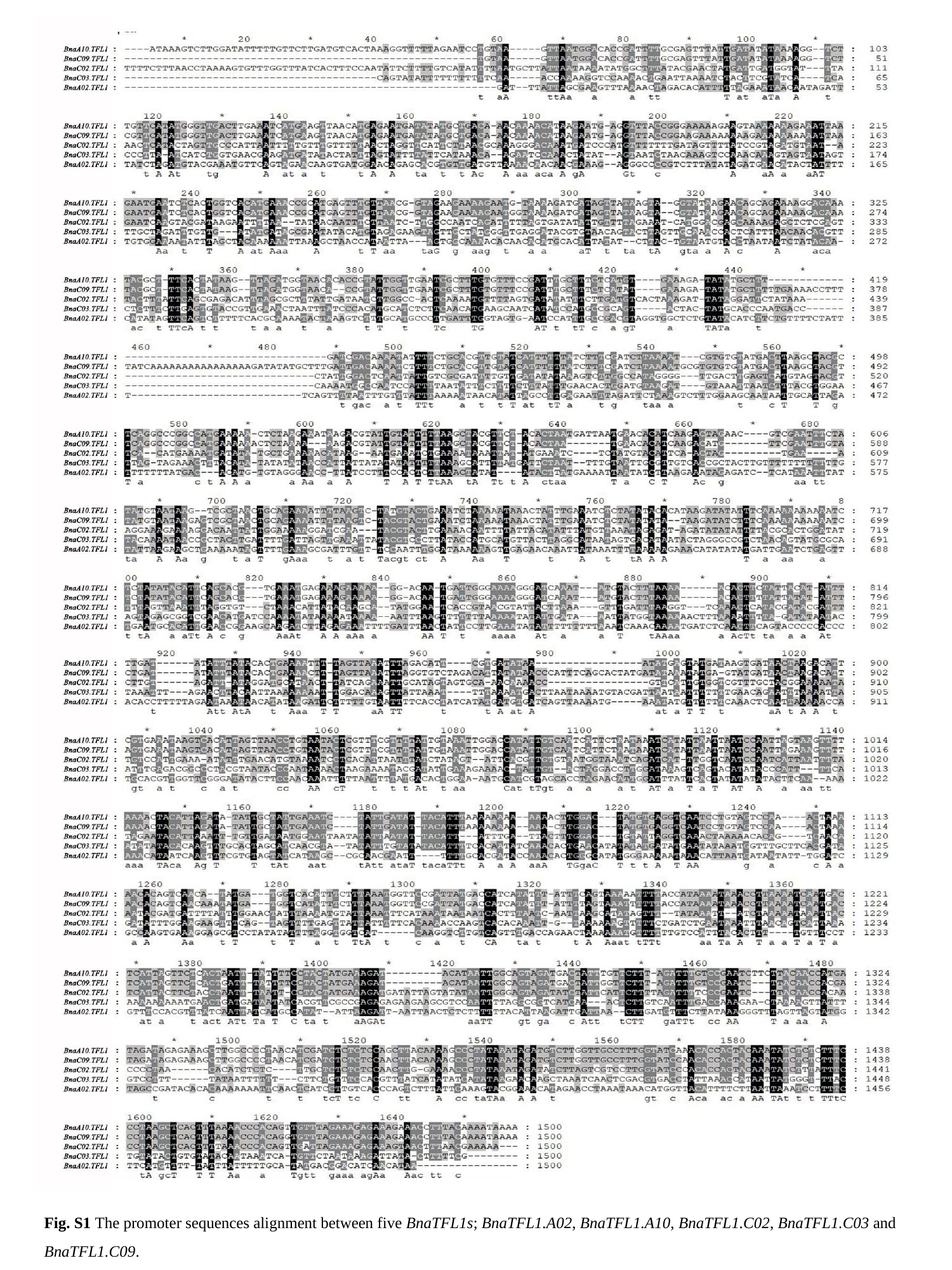

Fig. S1 The promoter sequences alignment between five BnaTFL1s; BnaTFL1.A02, BnaTFL1.A10, BnaTFL1.C02, BnaTFL1.C03 and BnaTFL1.C09.
